# Supplementary material for: Study of CdS/CdS Nanoparticles Thin Films Deposited by Soft Chemistry for Optoelectronic Applications
Source: Micromachines (Basel). 2023 May 31;14(6):1168. doi: 10.3390/mi14061168 (PMC10300819; doi:10.3390/mi14061168)
Supplement: Supplementary file 1 [file micromachines-14-01168-s001.zip › micromachines-2411821-supplementary.pdf]

micromachines

MDPI

Supplementary Materials

Study of CdS/CdS Nanoparticles Thin Films Deposited by Soft Chemistry for Optoelectronic Applications

Laura Aislinn Carrasco-Chavez [1], José F. Rubio-Valle [2], Abimael Jiménez-Pérez [1], José E. Martín-Alfonso [2,*] and Amanda Carrillo-Castillo [1,*]

[1] Institute of Engineering and Technology, Autonomous University of Ciudad Juarez, Juarez Chihuahua 32310, Mexico; al187107@alumnos.uacj.mx (L.A.C.-C.); abimael.jimenez@uacj.mx (A.J.-P.)
[2] Chemical Product and Process Technology Research Center (Pro²TecS), Department of Chemical Engineering and Materials Science, University of Huelva, 21071 Huelva, Spain; josefernando.rubio@diq.uhu.es
* Correspondence: jose.martin@diq.uhu.es (J.E.M.-A.); amanda.carrillo@uacj.mx (A.C.-C.); Tel.: +34-959-218204 (J.E.M.-A.); +52-1-656-688-4800 (A.C.-C.)

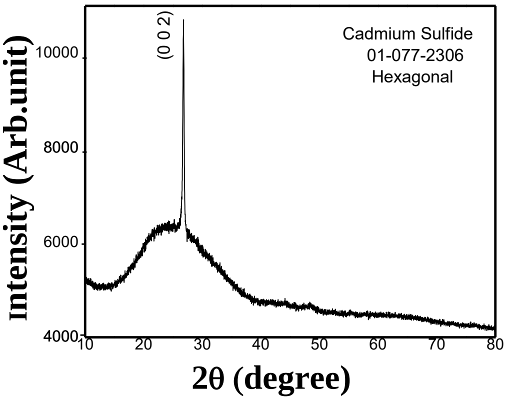

(**a**)

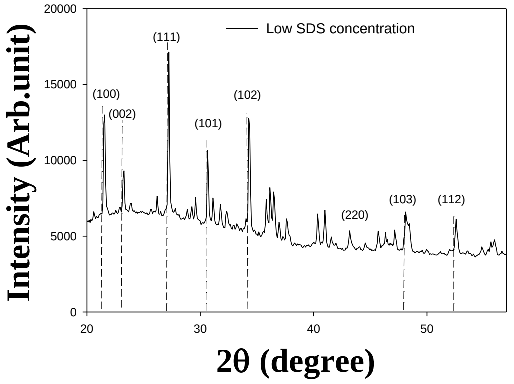

(**b**)

Figure S1. X-ray diffraction pattern reported for (**a**) CdS in two layers without annealing reported by (Palma-Soto et al. 2021) [39] and (**b**) CdS Nps as- synthetized (Carrillo et al. 2012) [47].
